# Parthenolide Relieves Pain and Promotes M2 Microglia/Macrophage Polarization in Rat Model of Neuropathy

**DOI:** 10.1155/2015/676473

**Published:** 2015-05-18

**Authors:** Katarzyna Popiolek-Barczyk, Natalia Kolosowska, Anna Piotrowska, Wioletta Makuch, Ewelina Rojewska, Agnieszka M. Jurga, Dominika Pilat, Joanna Mika

**Affiliations:** Department of Pain Pharmacology, Institute of Pharmacology Polish Academy of Sciences, Smetna 12, 31-343 Krakow, Poland

## Abstract

Neuropathic pain treatment remains a challenge because pathomechanism is not fully understood. It is believed that glial activation and increased spinal nociceptive factors are crucial for neuropathy. We investigated the effect of parthenolide (PTL) on the chronic constriction injury to the sciatic nerve (CCI)-induced neuropathy in rat. We analyzed spinal changes in glial markers and M1 and M2 polarization factors, as well as intracellular signaling pathways. PTL (5 *µ*g; *i.t.*) was preemptively and then daily administered for 7 days after CCI. PTL attenuated the allodynia and hyperalgesia and increased the protein level of IBA1 (a microglial/macrophage marker) but did not change GFAP (an astrocyte marker) on day 7 after CCI. PTL reduced the protein level of M1 (IL-1*β*, IL-18, and iNOS) and enhanced M2 (IL-10, TIMP1) factors. In addition, it downregulated the phosphorylated form of NF-*κ*B, p38MAPK, and ERK1/2 protein level and upregulated STAT3. In primary microglial cell culture we have shown that IL-1*β*, IL-18, iNOS, IL-6, IL-10, and TIMP1 are of microglial origin. Summing up, PTL directly or indirectly attenuates neuropathy symptoms and promotes M2 microglia/macrophages polarization. We suggest that neuropathic pain therapies should be shifted from blanketed microglia/macrophage suppression toward maintenance of the balance between neuroprotective and neurotoxic microglia/macrophage phenotypes.

## 1. Introduction

Despite advances in medicine and the systematic introduction of new drugs, the available treatment for neuropathic pain is still not satisfactory [1–3]. Recent data indicate a crucial role of glia activation in the development of neuropathic pain, which is now considered to be a neuroimmune disorder. There is a rapidly growing body of evidence indicating that signaling from microglia plays a relevant role in the pathogenesis of neuropathic pain [4–8]. The microglia rapidly became activated and mobilized to the site of injury where they initiate the release of effector molecules and recruitment of other immune cells. Macrophages from the peripheral circulation enter the CNS at the site of injury and contribute to secondary damage-phagocytose dead cells and clear tissue debris [9]. Unfortunately there is a lack of discriminating morphological or antigenic markers to identify the cellular origin of these two cell types at the site of injury. Several studies indicate that microglia/macrophage activation is a polarized process, which results in cells with either pro- or antinociceptive properties; however, the regulation of this phenomenon is still unknown [9, 10]. Polarization of microglia/macrophage is divided into two phases: the potentially neurotoxic M1 state called “classical activation” and the neuroprotective M2 state known as “alternative activation” [10, 11]. The phase M1 phenotype is characterized by proinflammatory factors with pronociceptive properties, for example, interleukin (IL-1*β*, IL-18, and IL-6) and inducible nitric oxide synthase (iNOS) [12–14]. The M2 phase is associated with the release of markers, which are anti-inflammatory factors with antinociceptive properties, such as IL-10 and tissue inhibitor of metalloproteinases 1 (TIMP1) [12–15]. In our work with microglial primary cell cultures [16] we reported that activated microglial cells are an important source of many nociceptive factors, like IL-1*β*, IL-6, IL-18, IL-1*α*, and IL-10. After injury spinal microglia result in the release of both algesic (IL-1*β*, IL-18, IL-6, and iNOS) and analgesic (IL-10 and TIMP1) mediators [5, 9, 16, 17]. PTL, sesquiterpene lactone occurring in the leaves of feverfew (*Tanacetum parthenium*), is considered to be the main component manifesting biological activity in the extracts of this perennial plant [18]. Infusions of feverfew or oral preparations containing PTL have been applied in patients to prevent migraine and rheumatic pain [18, 19]. In 2014, we published [20] that parthenolide (PTL) decreased allodynia and hyperalgesia after sciatic nerve injury.* In vitro* studies suggest that PTL affects some cellular pathways, for example, nuclear factor-kappa B (NF-*κ*B) [21–23], signal transducers and activators of transcription (STATs) [24], and MAP kinases [25, 26], but its influence on these factors under neuropathic pain is still unknown. The results of many studies suggest that the spinal activation of the p38, ERK1/2, NF-*κ*B, and STAT3 pathways plays a role in the pathogenesis of neuropathic pain [5, 27, 28] and contributes to the downstream activation of many nociceptive factors [5, 29–32].

Therefore, the aim of the present study was first to examine the influence of PTL on spinal glial cell activation in a rat neuropathic pain model (chronic constriction injury of the sciatic nerve (CCI)). We analyzed the protein levels of both the pronociceptive (IL-1*β*, IL-18, IL-6, and iNOS) and the antinociceptive (IL-10 and TIMP1) factors in the dorsal horn of the lumbar spinal cord in the CCI-exposed rats after PTL administration. Using primary microglial cell cultures we reported that those cells are an important source of analyzed factors. The next step of our work was to determine which signaling pathway is involved in PTL-changed balance between the pronociceptive and the antinociceptive factors characteristic for neuropathic pain.

## 2. Materials and Methods

### 2.1. Animals

Male Wistar rats (300–350 g) were housed in cages lined with sawdust under a standard 12/12 h light/dark cycle (lights on at 08:00 h), with food and water available* ad lib*. Care was taken to reduce the number of animals used. All experiments were performed according to the recommendations of IASP [33] and the NIH Guide for the Care and Use of Laboratory Animals and were approved by the II Local Bioethics Committee branch of the National Ethics Committee for Experiments on Animals based at the Institute of Pharmacology, Polish Academy of Sciences (Cracow, Poland).

### 2.2. Surgical Preparations

Chronic constriction injury (CCI) was produced in the rats by tying four ligatures around the sciatic nerve under sodium pentobarbital anesthesia (60 mg/kg; intraperitoneal (*i.p*.)). The* biceps femoris* and the* gluteus superficialis* were separated, and the right sciatic nerve was exposed. The ligatures (4/0 silk) were tied loosely around the nerve distal to the sciatic notch with 1 mm spacing until they elicited a brief twitch in the respective hind limb. The procedure has been described in detail by Bennett and Xie [34]. After the surgery, all rats developed long-lasting neuropathic pain symptoms such as allodynia and hyperalgesia. It was reported, by our group [35] as well as others researchers [36], that there are no significant differences between sham-operated and naive animals in pain thresholds used in neuropathic pain model. Therefore, to limit the number of animals in the present study we decided to use naive animals as a control group.

### 2.3. Intrathecal (*i.t.*) Injection

The rats were prepared for intrathecal (*i.t*.) injection by implanting catheters under pentobarbital anesthesia (60 mg/kg* i.p*.). The intrathecal catheter consisted of polyethylene tubing that was 12 cm long (PE 10, Intramedic; Clay Adams, Parsippany, NJ) with an outside diameter of 0.4 mm and a dead space of 10 *μ*L that had been sterilized by immersion in 70% (v/v) ethanol and fully flushed with sterile water before insertion. The rats were placed on a stereotaxic table (David Kopf Instruments, Tujunga, CA), and an incision was made in the atlantooccipital membrane. The catheter (7.8 cm of its length) was carefully introduced into the subarachnoid space at the rostral level of the spinal cord lumbar enlargement (L4-L5) according to the method of Yaksh and Rudy [37]. After the implantation, the first injection of 10 *μ*L of water was performed slowly and the catheter was tightened. One day after catheter implantation, the rats were monitored for physical impairments. Those showing motor deficits were excluded from further study. Animals were allowed a minimum 1 week of recovery after the surgery before the experiment began. Substances were injected slowly (1-2 min) in a volume of 5 *μ*L through the* i.t.* catheter and were followed by 10 *μ*L of water.

### 2.4. Behavioral Tests

#### 2.4.1. Tactile Allodynia (von Frey Test)

Allodynia was measured in the rats subjected to CCI by the use of an automatic von Frey apparatus (Dynamic Plantar Aesthesiometer cat.number 37400, Ugo Basile, Italy). The rats were placed in plastic cages with a wire net floor 5 min before the experiment. The von Frey filament (up to 26 g) was applied to the midplantar surface of the hind foot, and measurements were taken automatically [31].

#### 2.4.2. Hyperalgesia (Cold Plate Test)

Hyperalgesia was assessed using the cold plate test (Cold/Hot Plate Analgesia Meter number 05044 Columbus Instruments, USA) as has been described previously [6, 31]. The temperature of the cold plate was maintained at 5°C, and the cut-off latency was 30 s. The rats were placed on the cold plate, and the time until lifting of the hind foot was recorded. The injured foot was the first to react in every case.

### 2.5. Microglial Cell Cultures

Primary microglial cells cultures were prepared as has been previously described [20]. Cells were isolated from 1-day-old Wistar rats' cerebral cortices and were grown at 37°C and 5% CO_2_ in poly-L-lysine-coated 75 cm^2^ culture flasks in a culture medium DMEM/Glutamax/high glucose (Gibco, New York, USA) supplemented with 10% foetal bovine serum (Gibco, New York, USA), 100 U/mL penicillin, and 0.1 mg/mL streptomycin (Gibco, New York, USA). The culture medium was changed after 4 days. The loosely adherent microglial cells were recovered after 9 days by mild shaking and centrifugation and then were suspended in a culture medium and plated at a final density of 2 × 10^5^ cells onto 24 well plates. Adherent cells were incubated for 48 h in a culture medium before being used for the analyses. Cell specificity was determined using an antibody to OX-42 (a microglial marker) in cultures of primary microglia. Levels of mRNA for* C1q* (a microglial marker) and* GFAP* (an astroglial marker) were also investigated. Cultured primary microglia were more than 95% positive for OX-42 and* C1q*.

Primary microglial cell cultures were treated with vehicle (PBS) or LPS [100 ng/mL] (Lipopolysaccharide from* Escherichia coli* 0111:B4 (Sigma-Aldrich, Saint Louis, USA)) for 24 h for mRNA and protein analysis.

### 2.6. qRT-PCR Analysis

Cell samples were collected in Trizol Reagent (Invitrogen, Carlsbad, CA, USA) and RNA isolation was conducted according to Chomczynski's method [38]. The total RNA concentration was measured using a NanoDrop ND-1000 Spectrometer (Nano-Drop Technologies, Wilmington, DE, USA). Reverse transcription was performed on 500 ng of total RNA using Omniscript reverse transcriptase (Qiagen Inc.) at 37°C for 60 min. RT reactions were carried out in the presence of an oligo (dT16) primer (Qiagen Inc.). The cDNA was diluted 1 : 10 with H_2_O; for each reaction ~50 ng of cDNA synthesised from the total RNA of an individual sample was used for the qRT-PCR reaction. Analysis was performed using Assay-On-Demand TaqMan probes according to the manufacturer's protocol (Applied Biosystems) and the reactions were performed on an iCycler device (BioRad, Hercules). The following TaqMan primers were used: Rn01527838_g1 (HPRT, hypoxanthine guanine rat hypoxanthine guanine phosphoribosyl transferase); Rn00587558_m1 (TIMP1, metallopeptidase inhibitor 1); Rn00580432_m1 (IL-1beta, interleukin 1*β*); Rn00561420_m1 (IL-6, interleukin 6); Rn01422083_m1 (IL-18, interleukin 18); Rn00563409_m1 (IL-10, interleukin 10); and Rn00561646_m1 (iNOS, inducible nitric oxide synthase). The amplification efficiency for each assay was determined by running a standard dilution curve. The cycle threshold (Ct) values were calculated automatically using the iCycler IQ 3.0 software with the default parameters. Expression of the* Hprt1* transcript was quantified to control for variation in cDNA amounts. The abundance of RNA was calculated as 2^−(normalised threshold cycle)^.

### 2.7. Western Blot Analysis

Ipsi-dorsolateral L4–L6 spinal cords were collected from animals participating in behavioral test 6 h after vehicle or PTL administration at day 7 after CCI. The lysates from cell cultures and tissue were collected in RIPA buffer with protease and phosphatase inhibitor cocktails (Sigma-Aldrich) and cleared by centrifugation (14000 ×g for 30 min, 4°C). Protein concentration in the supernatant was determined using the BCA Protein Assay Kit (Sigma-Aldrich). Samples containing 14 *μ*g of protein were heated in a Laemmli Sample Buffer (4x): 250 mM Tris-HCl, pH 6.8, 4% LDS, 40% (w/v) glycerol, 0.02% bromophenol blue, and 15% *β*-mercaptoethanol (Bio-Rad) for 5 min at 95°C and resolved using SDS–PAGE on 4–20% polyacrylamide gels (Criterion TGX, Bio-Rad). Following the gel electrophoresis, proteins were transferred via electroblotting onto Immun-Blot PVDF membranes (Bio-Rad). After the transfer, membranes were blocked for 1 h at 25°C using 5% nonfat dry milk (Bio-Rad) in Tris-buffered saline with 0.1% Tween 20 (TBST). Next, the blots were incubated with the following anti-rat primary antibodies that had been diluted in a SignalBoost Immunoreaction Enhancer Kit (Calbiochem) for 24 h at 4°C: IBA1 (ProteinTech) 1 : 1000; GFAP (Novus Biologicals) 1 : 50000; IL-1*β* (Abcam) 1 : 1000; IL-6 (Invitrogen) 1 : 1000; iNOS (Sigma-Aldrich) 1 : 2000; IL-18 (R&D Systems) 1 : 1000; IL-10 (Invitrogen) 1 : 2000; TIMP1 (Novus Biologicals) 1 : 2000; p-p38 MAPK (Cell Signalling) 1 : 2000; p-ERK1/2 (Santa Cruz) 1 : 4000; p-NF-*κ*B (Santa Cruz) 1 : 2000; and p-STAT3 (Cell Signaling) 1 : 2000. After four 5-minute washes in TBST, blots were incubated with anti-rabbit, anti-mouse, or anti-goat secondary antibodies conjugated to horseradish peroxidase (HRP) at a dilution of 1 : 5000 in a SignalBoost Immunoreaction Enhancer Kit for 1 h at room temperature. After four additional 5-minute washes in TBST, immunocomplexes were detected using Clarity Western ECL Substrate (Bio-Rad) and visualized using a Fujifilm Luminescent Image Analyzer LAS4000 System. Blots were washed 2 times for 5 minutes each in TBS, stripped using Restore Western Blot Stripping Buffer (Thermo Scientific), washed 2 additional times for 5 minutes each in TBS, blocked, and reprobed with an antibody against GAPDH (Millipore) as an internal loading control at a dilution of 1 : 5000 in SignalBoost Immunoreaction Enhancer Kit. The relative levels of immunoreactivity were quantified densitometrically using Fujifilm Multi Gauge software.

### 2.8. Drug Administration

Parthenolide (PTL; Sigma-Aldrich, USA, 5 *μ*g/5 *μ*L;* i.t.*) was dissolved in 50% DMSO and preemptively administered* i.t.* 16 h and 1 h before CCI and then once daily for 7 days. The control groups received vehicle (50% DMSO) according to the same schedule. This method of PTL or vehicle administration was used throughout the work and is referred to as “repeated administration” in the text. Behavioral tests were conducted 30 min (von Frey test) and 35 min (cold plate test) after PTL administration.

### 2.9. Data Analysis

The behavioral data are presented as the mean ± SEM of 10–20 rats per group. The results of the experiments were statistically evaluated using one-way analysis of variance (ANOVA). All of the differences between the treatment groups were further analyzed with Bonferroni's post hoc tests. Significant differences in comparisons with the control group (naïve rats) are indicated by ^∗∗∗^ *P* < 0.001. Significant differences between the vehicle-treated CCI-exposed rats and the PTL-treated CCI-exposed rats are indicated by ^###^ *P* < 0.001.

The protein analyses were performed using Western blots in three groups: naïve, vehicle-treated CCI-exposed, and PTL-treated CCI-exposed rats. The results are presented as fold changes compared to the naïve rats in the ipsilateral dorsal lumbar spinal cord. The data are presented as the means ± SEM and represent the normalized averages derived from analyses of five to seven samples for each group performed with the Multi Gauge analysis program. Intergroup differences were analyzed using ANOVA followed by Bonferroni's multiple comparison tests. ^∗^ *P* < 0.05, ^∗∗^ *P* < 0.01, and ^∗∗∗^ *P* < 0.001 indicate significant differences compared to the naive rats. ^#^ *P* < 0.05, ^##^ *P* < 0.01, and ^###^ *P* < 0.001 indicate significant differences between the vehicle-treated and the PTL-treated CCI-exposed rats. The immunoblots shown are representative of 5–7 individual samples.

The results of the primary microglial cell cultures are presented as the fold change compared with the control group (vehicle-treated cells). The data are presented as the mean ± SEM. Intergroup differences were analysed with Student's *t*-test. ^∗^ *P* < 0.05, ^∗∗^ *P* < 0.01, and ^∗∗∗^ *P* < 0.001 indicate differences compared with the LPS-treated cells. The immunoblots shown are representative of 5–7 individual samples.

## 3. Results

### 3.1. Repeated PTL Administration Reduced Development of Neuropathic Pain in Rats

**Figure 1:**
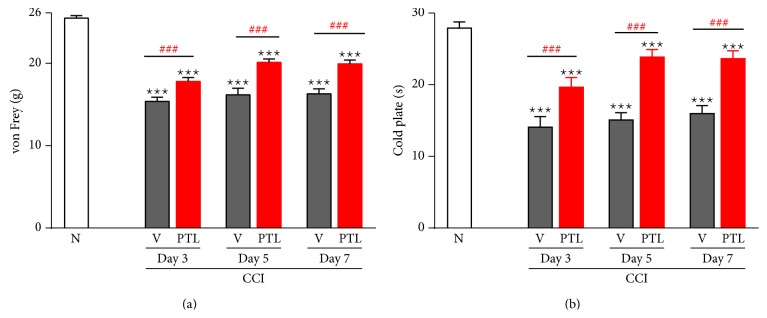
The effect of PTL on allodynia (von Frey test) and hyperalgesia (cold plate test) in CCI-exposed rats. Naïve rats do not show symptoms of neuropathy ((a) and (b)). In CCI-exposed rats, PTL (5 *μ*g/5 *μ*L) and vehicle were* i.t.* administered preemptively 16 h and 1 h before CCI and once daily over 7 days. The response to PTL was measured 30 minutes after administration by the von Frey test (a) and 35 minutes after administration by the cold plate test (b) 1 day before CCI and then on days 3, 5, and 7 after injury. Repeated PTL administration diminished the development of neuropathic pain. PTL significantly reduced mechanical allodynia (a) and thermal hyperalgesia (b) on days 3, 5, and 7 after CCI. The data are presented as the mean response ± SEM (10–20 rats per group). The results of the experiments were statistically evaluated using one-way analyses of variance (ANOVA). The differences between the treatment groups throughout the study were further analyzed with Bonferroni's post hoc tests. ^∗∗∗^ *P* < 0.001 indicate significant differences compared to naïve rats. ^###^ *P* < 0.001 indicate significant differences between vehicle-treated and PTL-treated CCI-exposed rats. N-naïve, V-vehicle, and PTL-parthenolide.

The control group (naïve) showed no response to mechanical stimuli (25.9 g ± 0.1) in the von Frey test (Figure 1(a)) on to thermal stimuli in the cold plate (30 s ± 0.2) test on days 3, 5, and 7 (Figure 1(b)). In the behavioral tests, all CCI vehicle-treated rats developed neuropathic pain symptoms. In the vehicle-treated CCI-exposed rats, allodynia in the ipsilateral paw was observed, as measured using the von Frey test on the 3, 5, and 7 days after ligation (15.37 g ± 0.5, 16.2 g ± 0.8, and 16.3 g ± 0.6, resp.) (Figure 1(a)) and the hyperalgesia as measured using the cold plate test (14.1 s ± 1.5, 15.1 s ± 1.0, and 16.0 s ± 1.1, resp.) (Figure 1(b)). Repeated treatment of PTL (5 *μ*g/5 *μ*L;* i.t.*) significantly attenuated allodynia in the ipsilateral paw at days 3, 5, and 7 after injury, as measured using von Frey test 30 min after PTL administration (17.8 g ± 0.5, 20.1 g ± 0.4, and 19.9 g ± 0.5 at days 3, 5, and 7 after CCI, resp.) (Figure 1(a)) and significantly decreased hyperalgesia, as measured using the cold plate test 35 min after PTL administration (19.7 s ± 1.4, 23.9 s ± 1.0, and 23.7 s ±1.0 at days 3, 5, and 7 after CCI, resp.) (Figure 1(b)).

### 3.2. Repeated PTL Administration Influenced Microglia/Macrophage Activation Level in the Spinal Cord, 7 Days after CCI

**Figure 2:**
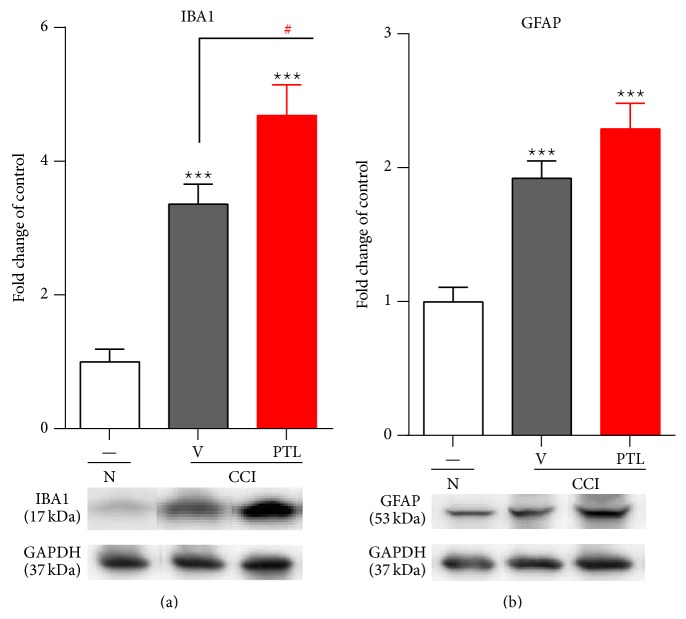
Repeated PTL administration influenced microglia/macrophage activation in the spinal cord level under neuropathic pain. Seven days after CCI in the ipsilateral dorsal spinal cord the protein level for IBA1 (a) and GFAP (b) were upregulated due to nerve injury. Repeated PTL administration increased the level of IBA1 (a), but the GFAP protein level was unchanged (b). The Western blot data are presented as the mean ± SEM and represent the normalized averages derived from analyses of 5–7 samples for each group. Intergroup differences were analyzed using ANOVA followed by Bonferroni's multiple comparison test. ^∗∗∗^ *P* < 0.001 indicate significant differences compared to naïve rats. ^#^ *P* < 0.05 indicate significant differences between vehicle-treated and PTL-treated CCI-exposed rats. N-naïve, V-vehicle, and PTL-parthenolide. The immunoblots shown are representative of 5–7 individual samples.

Seven days after CCI, in the lumbar dorsal spinal cord, we observed the upregulation of protein levels by 337% for IBA1 (a microglial/macrophage marker) and 193% for GFAP (an astrocyte marker) in the vehicle-treated CCI-exposed rats compared with the naïve animals (Figures 2(a) and 2(b), resp.). Repeated administration of PTL increased the levels of microglial/macrophage activation marker to 470% compared with the vehicle-treated CCI-exposed rats (Figure 2(a)) but did not cause significant changes to the levels of astrocyte marker (Figure 2(b)).

### 3.3. Repeated PTL Administration Influenced Spinal Pronociceptive (IL-1*β*, IL-6, IL-18, and iNOS) and Antinociceptive Factors (IL-10 and TIMP1), 7 Days after CCI

**Figure 3:**
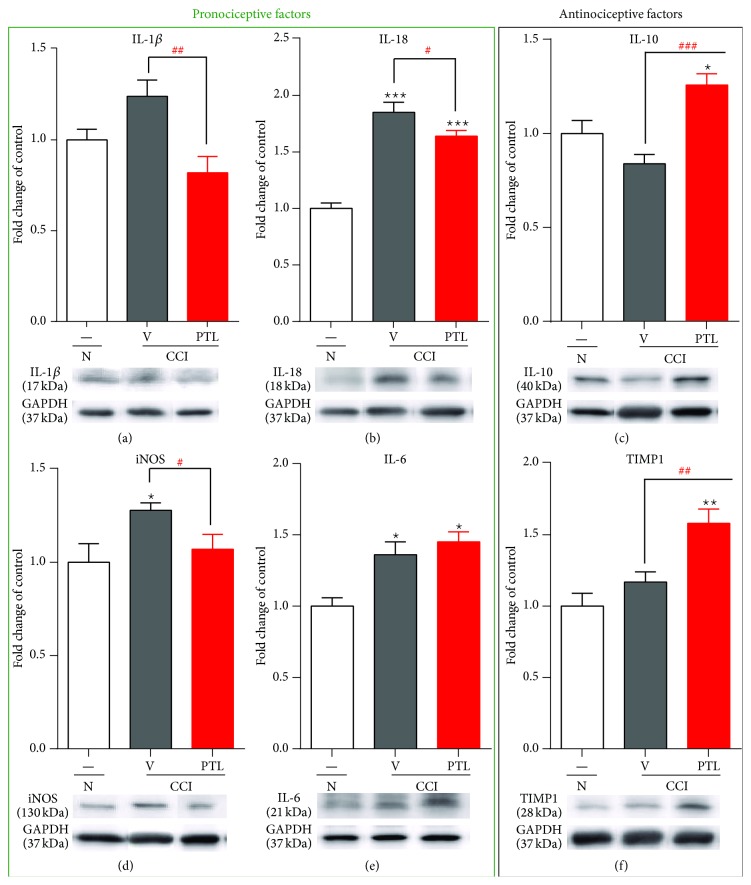
Effects of PTL on the protein levels of pronociceptive (IL-1*β*, IL-18, iNOS, and IL-6) and antinociceptive (IL-10 and TIMP1) factors in the spinal cord 7 days after CCI. The nerve injury caused increased IL-18 (b), iNOS (d), and IL-6 (e) protein levels, but the IL-1*β* (a) protein level was unchanged after CCI. Chronic PTL administration diminished protein levels of IL-1*β* (a), IL-18 (b), and iNOS (d) but did not change IL-6 (e) in the dorsal part of the lumbar spinal cord 7 days after injury. The protein level of IL-10 (c) and TIMP1 (f) was unchanged in the spinal cord following CCI, but repeated administration of PTL significantly increased IL-10 (c) and TIMP 1 (f) proteins compared to those of CCI-exposed rats without PTL treatment. The data are presented as the mean ± SEM of 5–7 samples from each group. Intergroup differences were analyzed using Bonferroni's multiple comparison test. ^∗^ *P* < 0.05, ^∗∗^ *P* < 0.01, and ^∗∗∗^ *P* < 0.001 indicate significant differences compared to naïve rats. ^#^ *P* < 0.05, ^##^ *P* < 0.01, and ^###^ *P* < 0.001 indicate differences between vehicle-treated and PTL-treated CCI-exposed group. N-naïve, V-vehicle, and PTL-parthenolide. The immunoblots shown are representative of 5–7 individual samples.

The protein levels of IL-1*β*, IL-18, IL-6, iNOS, IL-10, and TIMP1 in the ipsilateral dorsal spinal cord (L4–L6) were examined using Western blot analysis. Following CCI, the regulation of IL-1*β* protein level (amounting to 124%) was not statistically significant in the spinal cord (Figure 3(a)). PTL administration significantly downregulates IL-1*β* (by 66%) protein levels in the CCI-exposed rats (Figure 3(a)). A strong upregulation of 136% for IL-18 protein level was observed (Figure 3(b)) following CCI and PTL significantly prevented the injury-induced increase (by 87%) of IL-18 protein levels (Figure 3(b)). Upregulation of iNOS (Figure 3(d)) protein amounting to 128% was observed in the CCI-exposed rats. This level was lower in the animals receiving PTL than in the animals following nerve injury (by 84% Figure 3(d)). The significant increase of IL-6 protein level (by 136%) (Figure 3(e)) was observed following CCI, and PTL administration did not affect the protein levels (Figure 3(e)).

No changes in the level of IL-10 (Figure 3(c)) or TIMP1 (Figure 3(f)) proteins were observed in the CCI-exposed rats. Repeated PTL administration caused significant increase in IL-10 (by 50% Figure 3(c)) and TIMP1 (by 35% Figure 3(f)) protein levels compared to those in the rats following nerve injury.

### 3.4. Repeated PTL Administration Inhibited Spinal p-NF-*κ*B, p-p38, and p-ERK1/2 and Enhanced p-STAT3, 7 Days after CCI

**Figure 4:**
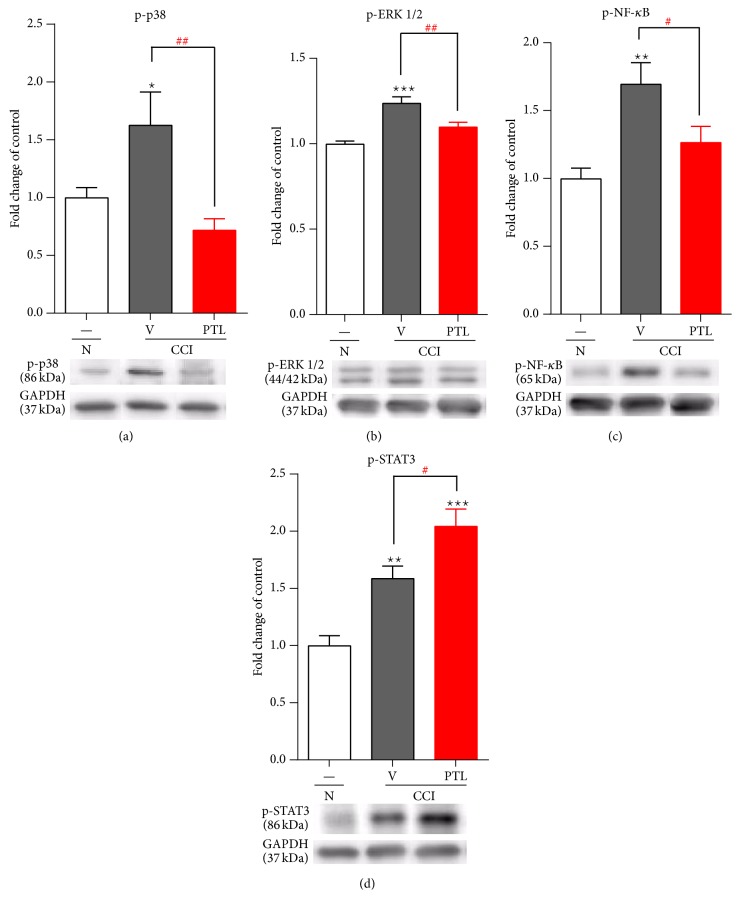
Effects of PTL on the protein levels of p-p38, p-ERK1/2, p-NF-*κ*B, and p-STAT3 in the spinal cord 7 days after CCI. Seven days after CCI in the lumbar dorsal cord a significant increase of p-p38 (a) and p-ERK1/2 (b) proteins levels was observed. Repeated administration of PTL reduced the upregulation of p-p38 (a) and p-ERK 1/2 (b) protein levels in comparison with those of CCI-exposed rats. Similarly, seven days after CCI in the ipsilateral dorsal spinal cord, p-NF-*κ*B (c) protein level was significantly upregulated by nerve injury and repeated PTL administration inhibited the level of p-NF-*κ*B (c). At day 7 after CCI, strong upregulation of p-STAT3 was observed (d). Chronic PTL-treatment caused enhancement of p-STAT3 (d). The Western blot data are presented as the mean ± SEM and represent the normalized averages derived from analyses of 5–7 samples for each group. Intergroup differences were analyzed using ANOVA followed by Bonferroni's multiple comparison test. ^∗^ *P* < 0.05, ^∗∗^ *P* < 0.01, and ^∗∗∗^ *P* < 0.001 indicate significant differences compared to naïve rats. ^#^ *P* < 0.05 and ^##^ *P* < 0.01 indicate significant differences compared with the vehicle-treated CCI-exposed rats. N-naïve, V-vehicle, and PTL-parthenolide. The immunoblots shown are representative of 5–7 individual samples.

The level of proteins p-NF-*κ*B, p-p38, and p-ERK 1/2 in the ipsilateral dorsal spinal cord (L4–L6) were examined using Western blot analysis. The p-p38 (by 163%) and p-ERK1/2 (by 124%) protein levels were increased in the vehicle-treated CCI-exposed rats (Figures 4(a) and 4(b), resp.). Repeated PTL administration prevents the upregulation of p-p38 (by 44%) and p-ERK (by 89%) protein levels (Figures 4(a) and 4(b), resp.). The p-NF-*κ*B protein level was upregulated (by 170%) compared with that in the naïve animals (Figure 4(c)). Repeated PTL administration significantly prevented upregulation of p-NF-*κ*B level (by 25%) in the spinal cord 7 days after CCI (Figure 4(c)). Upregulation of p-STAT3 protein by 159% in the vehicle-treated CCI-exposed rats compared with that of the naïve animals was observed (Figure 4(d)). Repeated administration of PTL increased the level of p-STAT3 compared to that of the vehicle-treated neuropathic rats (by 28%).

### 3.5. The Influence of LPS Stimulation on mRNA and Protein Levels of Pro- (IL-1*β*, IL-18, IL-6, and iNOS) and Antinociceptive (IL-10 and TIMP1) Factors in Microglial Cells:* In Vitro* Studies

**Figure 5:**
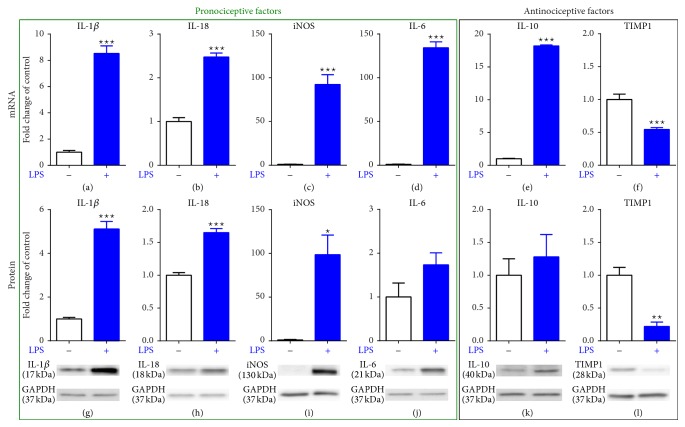
Effects of LPS on the mRNA and protein level of pronociceptive (IL-1*β*, IL-18, IL-6, and iNOS) and antinociceptive (IL-10 and TIMP1) factors in the primary rat microglial cell cultures. Samples were analyzed 24 hours after stimulation of cells with LPS. Upregulation of mRNA for* IL-1β* (a),* IL-18* (b),* iNOS* (c),* IL-6* (d),* IL-10* (e), and downregulation of mRNA for* TIMP1* (f) were observed after LPS treatment. The protein level of IL-1*β* (g), IL-18 (h), and iNOS (i) were significantly upregulated and of TIMP1 (l) was downregulated after LPS stimulation. Protein level of IL-6 and IL-10 remained unchanged after LPS administration. The qRT-PCR and Western blot data are presented as the mean ± SEM and represent the normalized averages derived from analyses of 3–10 samples for each group. Statistical analysis was performed using *t* test, ^∗^ *P* < 0.05, ^∗∗^ *P* < 0.01, and ^∗∗∗^ *P* < 0.001 indicate significant differences compared to vehicle-treated cells. LPS: lipopolysaccharide. The immunoblots shown are representative of 3–5 individual samples.

The mRNA level of* IL-1β, IL-18, IL-6, iNOS, IL-10*, and* TIMP1* in vehicle- and LPS-treated microglial cell was analyzed. 24 h after the administration of LPS [100 ng/mL] a significant increase in secretion of all pronociceptive factors (*IL-1β, IL-18, IL-6,* and* iNOS*) (Figures 5(a), 5(b), 5(c), and 5(d), resp.; upper panel) and antinociceptive IL-10 (Figure 5(e), upper panel) was observed. The level of* TIMP1* (Figure 5(f), upper panel) was significantly downregulated after LPS treatment compared to vehicle-treated cells.

The protein levels of IL-1*β*, IL-18, IL-6, iNOS, IL-10, and TIMP1 in LPS-treated microglial cells were examined using Western blot analysis. A significant increase in the protein level of pronociceptive (IL-1*β*, IL-18, and iNOS) factors (Figures 5(g), 5(h), and 5(i), resp.; lower panel) after 24 h LPS [100 ng/mL] was detected. LPS did not influence the protein level of IL-6 and IL-10 (Figures 5(j) and 5(k), resp.; lower panel). The protein level of TIMP1 was significantly downregulated after 24 h LPS treatment compared to vehicle-stimulated cells (Figure 5(l), lower panel).

## 4. Discussion

Several studies have investigated the possible therapeutic opportunities to intervene in the development and maintenance of neuropathy. Our behavioral studies have shown that PTL reduced the allodynia and hyperalgesia on days 3, 5, and 7 after CCI, which is consistent with our previously published results [20]. Many studies, including ours, have shown glia activation under neuropathy [4, 5, 39–41]. We have already published [42] that spinal level, the gene expression for NK-cells (CD335), T-cells (Cd3g, Cd3e, Cd3d, CD4, and CD8), and B-cells (CD19) markers, is unchanged after CCI, in contrast to CD40 (a monocyte marker) which is strongly upregulated after injury. Similarly, in the present paper, we observed spinal increase of microglia/macrophages (IBA1) and astroglia (GFAP) markers at day 7 after CCI. In the literature, it has been demonstrated that downregulation of microglial activation, for example, by minocycline, diminished neuropathic pain [4, 6, 16, 17, 43–45]. Surprisingly, chronic administration of PTL, which exhibits an analgesic effect, upregulated microglial/macrophage activation. It is known that, at the site of injury, microglia/macrophages promote both the damage and repair of a tissue. Such divergent actions may be caused by subsets of those cells in the injured tissue: “classically activated” proinflammatory (M1) or “alternatively activated” anti-inflammatory (M2) cells. Several studies report that activated microglia/macrophages produce various pronociceptive cytokines (e.g., IL-1*β*, IL-6, and IL-18) and other factors (e.g., iNOS) that are cytotoxic and therefore contribute to secondary tissue damage, initiate neuroinflammation, neuronal cell death, and demyelination [16, 46–49]. In our primary microglial cell culture studies we have shown that all analyzed factors are produced by microglia.

iNOS is one of the most interesting M1 microglial/macrophage polarization markers [50]. Nitric oxide (NO)/iNOS has been implicated in the pathology of neuropathic pain, and it is generated by nonneuronal cells [31, 51–53]. In the study of Possel et al. [54] the authors demonstrated, by immunohistochemistry, that localization of iNOS expression is restricted to microglial cells. In our* in vitro* study we confirmed that microglial cells are the source of iNOS and LPS treatment upregulates expression of this pronociceptive factor, which is in agreement with other studies [55]. Our previous studies have shown that minocycline decreased the protein level of iNOS after CCI [31]. In the present paper, we have investigated whether PTL administration prevented the iNOS upregulation induced by nerve injury. However the effects of PTL are parallel to the upregulation of microglial/macrophage cell activation; so, in our opinion, it is associated with microglia/macrophage M2 polarization. IL-1*β* is another marker of microglial/macrophage M1 polarization. Intrathecal administration of this cytokine has been demonstrated to have algesic properties [56]. In rat model of spinal cord inflammation Clark et al. [57] demonstrated that spinal microglia activation is correlated with IL-1*β* secretion. Selenica et al. [58] revealed, using immunohistochemistry evaluation, that, in CCL2-injected mice, IL-1*β* positive cells displayed M1 microglial phenotype. In Parkinson's disease model immunohistochemistry also revealed colocalization of IL-1*β* with Iba-1 in CNS. Our* in vitro* study indicates the presence of IL-1*β* and strong upregulation after LPS stimulation as it was shown in other studies [14, 16]. Moreover, the phosphodiesterase inhibitor propentofylline reversed pronociceptive effects of IL-1*β* and normalized CCI-induced hypersensitivity [59]. IL-18 is a pronociceptive cytokine that is known to be of microglia/macrophage origin and to potentiate hyperalgesia [60–62]. Miyoshi et al. [60], using immunohistochemistry technique, showed that IL-18 is colocalised with Iba-1. The authors also revealed that nerve injury upregulated spinal IL-18 in microglia but not in neurons or astrocytes, as it was shown by double immunostaining with Iba-1, NeuN, and GFAP markers, respectively. For the first time we demonstrated the production of both mRNA and protein IL-18 by nonstimulated and LPS-treated microglial cells using primary cell cultures, which correspond well with results obtained from microglial cell line [63]. In the present study, we observed upregulation of IL-18 after sciatic nerve injury, which is correlated with microglial/macrophage cell activation. Interestingly, after PTL administration, we observed decrease of IL-1*β* and IL-18 protein level with a concomitant increase of microglia/macrophage activation, which suggests that this drug can change polarization of those cell types. Juliana et al. [64] reported that parthenolide inhibited the activity of inflammasomes in primary macrophage cell cultures by directly inhibiting caspase-1, which led to the downregulation of IL-1*β* and IL-18 level. Those results elucidate the molecular mechanism for the anti-inflammatory action of parthenolide in macrophages and correspond well with our data.

Another cytokine analyzed in present study is IL-6, which is considered to be a pleiotropic cytokine with pronociceptive and antinociceptive properties [65–67], but its role in neuropathy remains unclear. It is well documented that various CNS cell types can produce IL-6 but the astrocyte likely plays a dominant role [68, 69]. In 2005 Kaplin and collaborators [69] demonstrated that at the spinal cord level astrocytes are the main source of IL-6, while microglia and infiltrating immune cells exhibited less robust staining. Orihuela et al. [70] have shown the upregulation of* IL-6* mRNA induced by LPS in BV2, a microglial cell line, which was associated with M1 microglial phenotype. Those data are in agreement with our results. In the present study we observed similar to others an upregulation of IL-6 after nerve injury [16, 71, 72]; however it was not affected by PTL. Those data correlate well with our results presented in current study, while PTL did not change GFAP and IL-6 upregulated after CCI level, but this issue needs future investigation.

The balance of immune response is maintained by antinociceptive factors, which serve as negative-feedback regulators. In our studies, we examined the levels of IL-10 and TIMP1, which have a potentially neuroprotective effect and are specific to the M2 activation state of microglia/macrophages [12–15]. IL-10 inhibits the release of IL-1*β* and IL-6 [73, 74] and plays role in neuropathy [75]. Using immunohistochemistry Pisanu et al. [76] demonstrated expression of IL-10 within Iba-1-positive cells in CNS. TIMP1 was the second antinociceptive factor analyzed in the present study; TIMP1 is an endogenous tissue inhibitor of MMP-9, which is highly effective in attenuating allodynia [77]. We have previously shown that mRNA for TIMP1 is upregulated in neuropathy [16, 78] and it is also produced by microglia/macrophages [15, 16, 79, 80]. In the study of Wu et al. [80] the OX42-positive cells purified from the injured spinal cord appeared to have highly increased level of TIMP1. The protein for TIMP1 was also found in a murine microglial cell line (BV2) [81]. In our present study we have shown, for the first time, using primary cell cultures, that microglia are a source of TIMP1 and its expression is downregulated after LPS treatment. In the present paper, we did not observe any changes in IL-10 or TIMP1 protein levels in the CCI-exposed rats; however, after repeated administration of PTL, the levels of both factors were significantly increased. These data indicated that chronic administration of PTL not only prevented the upregulation of pronociceptive factors but also increased antinociceptive factors. Lacraz et al. [79] have shown that TIMP1 level is dose-dependently upregulated by IL-10 in macrophages. Those data explain upregulation of IL-10 and TIMP1 after PTL administration, which is correlated with microglia/macrophages activation.

To better understand the mechanism of PTL action, we investigated intracellular pathways that may be involved in its effects. All examined signaling pathways play a crucial role in development and maintenance of neuropathic pain and are involved in regulation of expression of analyzed pro- and antinociceptive mediators. The NF-*κ*B signaling pathway is one of the most important pathways and regulates many physiological processes and plays a key role in regulating the immune response. Activation of the NF-*κ*B family of transcription factors induces many of the pronociceptive factor genes (among others IL-1*β*, IL-18, IL-6, and iNOS) [82–84]. Pathophysiological states, such as neuropathic pain, are a result of a constitutive activity of NF-*κ*B, mainly heterodimer p50/p65 [85]. It is believed that inhibition of the NF-*κ*B signaling pathway is an important mechanism responsible for the anti-inflammatory properties of PTL [23].* In vitro* studies suggest that PTL may directly inactivate [21] or indirectly inactivate NF-*κ*B through degradation and phosphorylation of inhibitor I*κ*B*α* (IKK) [22, 23]. In the present study, we have shown that chronic PTL-treatment significantly prevented the upregulation of the phosphorylated NF-*κ*B protein level after CCI, which is consistent with decrease of analyzed pronociceptive factors.

Mitogen-Activated Protein Kinases (MAPKs) are other important molecules in cell signaling and are of particular interest in the development of neuropathic pain. In our present study, we analyzed two members of the MAPK family: p38 and ERK1/2. It is well documented that p38 plays a role in neuropathic pain, and this effect is correlated with microglia activation [16, 30, 32, 86, 87]. ERK1/2 has been linked to signal transduction cascades that also regulate neuropathic pain [20, 88]. Our results show an increase in protein levels of p-p38 and p-ERK1/2 under neuropathic pain, which is in agreement with the findings of others [25, 26, 29, 32, 86, 87, 89, 90]. In 2002, Uchi et al. [26] and Fiebich et al. [25] suggested that parthenolide has an inhibitory effect on the activity of p38 in monocyte-derived dendritic cells and ERK1/2 in rat primary microglial cells. We have shown for the first time under neuropathic pain conditions that chronic PTL treatment prevented the CCI-induced upregulation of both p-p38 and p-ERK1/2 proteins level as measured 7 days after nerve injury, which is reflected in a downregulated level of pronociceptive factors.

During neuropathy, activation of proteins from the STAT pathway is observed. In the present study, we also report an increase in STAT3 after nerve injury and that PTL administration causes an upregulation of the active STAT3 form. STAT3 transcription factor plays a crucial role in inflammatory reactions being stimulated by a number of cytokines and regulating the expression of many proteins that are involved in inflammation [55, 91]. IL-6 and IL-10 are STAT3 regulators [91]. As previously mentioned, IL-6 has dual effects on nociceptive processing; in contrast, IL-10 has strong antinociceptive properties, and STAT3 is essential for the function of both interleukins [92]. In 2014, Przanowski et al. [55] showed that BV2 microglial cells transfected with constructs encoding constitutively active STAT3 produced high levels of IL-6 and IL-10. These data support our hypothesis that PTL up-regulates STAT3, which leads to increased microglia-derived IL-10 level. In addition, it is believed that TIMP1 expression can be regulated by STAT3 [93, 94]. It is thought that STAT3 directly contributes to the high level of TIMP1 [94]. Our results suggest that there is a possible correlation between STAT3 activation and increased levels of TIMP1. In 2013, Koscsó et al. [15] showed that IL-10-induced TIMP1 expression is correlated with upregulation of STAT3 signaling pathway and M2 macrophage activation, which is in agreement with our results. Thus, it appears that the STAT3 signaling pathway plays an important role in the M2 polarization of microglia/macrophage, leading to potentially neuroprotective “alternative activation” under neuropathic pain. This issue needs to be studied in the future.

## 5. Summary

**Figure 6:**
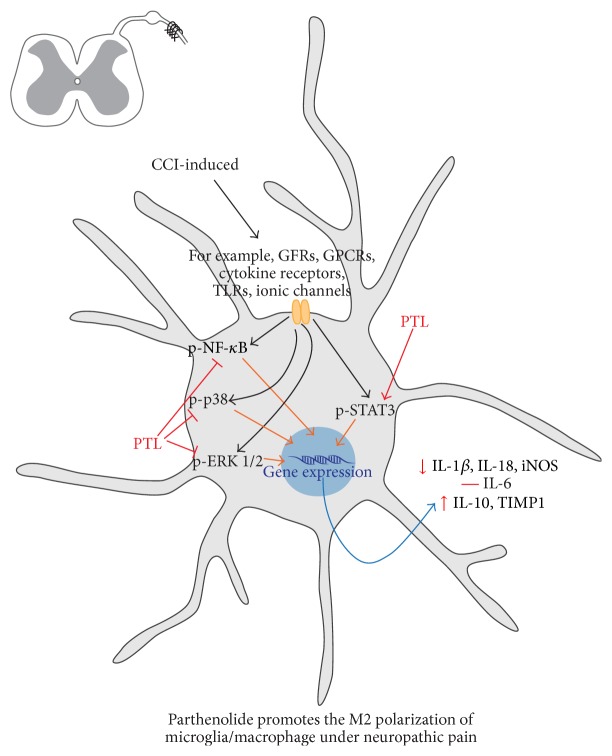
The possible influence of PTL on microglial/macrophage cell polarization. The extent of neuroinflammation observed under neuropathic pain depends on the bidirectional interactions between neurons and immune cells. As was suggested by many authors microglia/macrophages are among the main effector cells of the inflammatory response that takes place on the spinal cord level after injury [9, 42]. Therefore, it seems that targeting microglial signaling might lead to more effective treatment of neuropathic pain.* In vitro* evidence indicates that M1 microglia/macrophages can directly induce neuronal death [95, 96]. The activation of spinal microglia and infiltration of peripheral macrophages in the spinal cord can result in the release of both algesic (IL-1*β*, IL-18, IL-6, and iNOS) and analgesic (IL-10 and TIMP1) mediators during neuropathic pain [42]. Polarization of microglia/macrophage can strongly influence nociceptive transmission in neurons [7, 97]. It is known that polarization of microglia/macrophage is divided into two phases: potentially neurotoxic M1, which is characterized by pronociceptive factors, for example, IL-1*β*, IL-18, IL-6, iNOS, and neuroprotective M2 state, which is associated with release of antinociceptive markers, such as IL-10 and TIMP1 [12–15]. The results of our studies suggest that by direct or indirect mechanisms parthenolide promotes the M2 polarization of microglia/macrophage. In the present study, we have shown for the first time that chronic PTL treatment prevented the upregulated protein levels of pronociceptive IL-1*β*, IL-18, iNOS, and also potentiated antinociceptive IL-10 and TIMP1 in CCI-exposed rats. Simultaneously, PTL affects directly and/or indirectly intracellular pathways, which play a key role in regulating the immune response: NF-*κ*B, p38, ERK1/2, and STAT3. PTL induced reduction of NF-*κ*B, p38, ERK1/2, and potentiated STAT3 activation. These molecular actions of PTL might be responsible for its analgesic effects during neuropathy and need future investigation. (PTL: parthenolide, IL: interleukin, iNOS: inducible nitric oxide synthase, TIMP: tissue inhibitor of metalloproteinases, NF-*κ*B: nuclear factor-kappa B, ERK1/2: extracellular signal-regulated kinase 1/2, STAT: signal transducers and activators of transcription, GFRs: growth factor receptors, GPCRs: G protein–coupled receptors, TLRs: Toll-like receptors).

It seems that targeting microglia/macrophages signaling might lead to better understanding of neuropathic pain mechanisms and may give us clues for future therapeutic strategy. Glial activation and increased spinal pronociceptive factors strongly influence on neuronal transmission; therefore they are crucial in the development and maintenance of neuropathic pain. Recent studies suggest that the microenvironment of the spinal cord after injury favors M1 polarization with only a transient appearance of M2 microglia/macrophages. Optimal management of neuropathic pain is a major clinical challenge. Reducing an excessive or prolonged M1 state and enhancing M2 microglia/macrophage polarization may be a desirable therapeutic goal in neuropathic pain treatment. The new strategy for neuropathic pain therapy is to stimulate endogenous antinociceptive factors, which is more physiological than completely abrogating pronociceptive machinery. Our results suggests that PTL by direct or indirect mechanism promotes M2 microglia/macrophage polarization and attenuates neuropathic pain behavior (Figure 6;  supplementary chart in Supplementary Material available online at http://dx.doi.org/10.1155/2015/676473). Summing up, our results suggest that neuropathic pain therapies should be shifted from blanketed microglia/macrophage suppression toward maintenance of the balance between neuroprotective and neurotoxic microglia/macrophage phenotypes.

## Highlights

Repeated intrathecal administration of PTL in CCI-exposed rats is as follows:

- decreased neuropathic pain symptoms but enhanced microglia/macrophage activation,
- diminished the spinal protein level of pronociceptive factors IL-18, iNOS, and IL-1*β*,
- increased the spinal protein level of antinociceptive factors IL-10 and TIMP1,
- increased p-STAT3 and diminished p-NF-*κ*B, p-p38MAPK, and p-ERK1/2 protein levels.

## Supplementary Material

The graphical abstract of the analgesic effects of parthenolide (PTL) and associated changes in the glial cells, pro- and antinociceptive factors, and correlated signaling pathways activation at day 7 after CCI.
Repeated intrathecal administration of PTL in CCI-exposed rats is as follows:
(i) decreased neuropathic pain symptoms but enhanced microglia/macrophage activation.
(ii) diminished the spinal protein level of pronociceptive factors IL-18, iNOS, and IL-1beta.
(iii) increased the spinal protein level of antinociceptive factors IL-10 and TIMP1.
(iv) increased p-STAT3 and diminished p-NF-κB, p-p38, and p-ERK1/2 protein levels.

## Abbreviations

CCI: Chronic constriction injury
IBA1: Ionised calcium-binding adapter molecule 1
MAPK: Mitogen-activated protein kinase
NF-*κ*B: Nuclear factor *κ*B
PTL: Parthenolide
STAT3: Signal transducer and activator of transcription 3
TIMP1: Metallopeptidase inhibitor 1.
