# Supplementary material for: Parthenolide Relieves Pain and Promotes M2 Microglia/Macrophage Polarization in Rat Model of Neuropathy
Source: Neural Plast. 2015 May 18;2015:676473. doi: 10.1155/2015/676473 (PMC4452088; doi:10.1155/2015/676473)
Supplement: Supplementary file 1 — The graphical abstract of the analgesic effects of parthenolide (PTL) and associated changes in the glial cells, pro- and antinociceptive factors, and correlated signaling pathways activation at day 7 after CCI. Repeated intrathecal administration of PTL in CCI-exposed rats is as follows: (i) decreased neuropathic pain symptoms but enhanced microglia/macrophage activation. (ii) diminished the spinal protein level of pronociceptive factors IL-18, iNOS, and IL-1beta. (iii) increased the spinal protein level of antinociceptive factors IL-10 and TIMP1. (iv) increased p-STAT3 and diminished p-NF-κB, p-p38, and p-ERK1/2 protein levels. [file 676473.f1.pdf]

Analgesic effect of parthenolide (PTL) and associated changes in the glial cells, pronociceptive and antinociceptive factors, and correlated signaling pathways activation at 7 days, after CCI

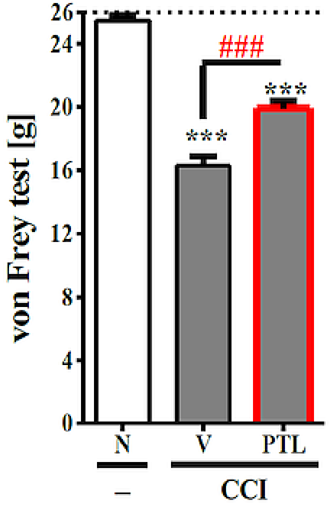

IPSILATERAL DORSAL HORN OF THE LUMBAR SPINAL CORD												
i.t.	GLIA MARKERS		PRONOCICEPTIVE FACTORS				ANTINOCICEPTIVE FACTORS		PROTEIN KINASES		TRANSCRIPTION FACTORS	
	IBA1	GFAP	IL-1β	IL-6	IL-18	iNOS	IL-10	TIMP1	p-p38	ERK1/2	NF-κB	p-STAT3
V-CCI	↑↑↑	↑↑↑	—	↑	↑↑↑	↑	—	—	↑	↑↑↑	↑↑	↑↑
PTL-CCI	↑	—	↓↓	—	↓	↓	↑↑↑	↑↑	↓↓	↓↓	↓	↑
